# ^18^F-FDG-PET-MRI for the assessment of acute intestinal graft-versus-host-disease (GvHD)

**DOI:** 10.1186/s12885-021-08748-x

**Published:** 2021-09-10

**Authors:** Wolfgang Roll, Philipp Schindler, Max Masthoff, Rebecca Strotmann, Jörn Albring, Christian Reicherts, Matthias Weckesser, Benjamin Noto, Matthias Stelljes, Michael Schäfers, Georg Evers

**Affiliations:** 1 Department of Nuclear Medicine, University Hospital Münster, Albert-Schweitzer-Campus 1, Building A1, 48149 Münster, Germany; 2 Department of Radiology, University Hospital Münster, Münster, Germany; 3 Department of Medicine A, Hematology, Oncology and Pulmonary Medicine, University Hospital Münster, Münster, Germany; 4 European Institute for Molecular Imaging, University of Münster, Münster, Germany

**Keywords:** GvHD, PET-MRI, Inflammation, FDG

## Abstract

**Background:**

Graft versus host disease (GvHD) is a frequent complication of allogeneic stem cell transplantation (alloSCT), significantly increasing mortality. Previous imaging studies focused on the assessment of intestinal GvHD with contrast-enhanced MRI/CT or ^18^F-FDG-PET imaging alone. The objective of this retrospective study was to elucidate the diagnostic value of a combined ^18^F-FDG-PET-MRI protocol in patients with acute intestinal GvHD.

**Methods:**

Between 2/2015 and 8/2019, 21 patients with acute intestinal GvHD underwent ^18^F-FDG-PET-MRI. PET, MRI and PET-MRI datasets were independently reviewed. Readers assessed the number of affected segments of the lower gastrointestinal tract and the reliability of the diagnosis on a 5-point Likert scale and quantitative PET (SUVmax, SUVpeak, metabolic volume (MV)) and MRI parameter (wall thickness), were correlated to clinical staging of acute intestinal GvHD.

**Results:**

The detection rate for acute intestinal GvHD was 56.8% for PET, 61.4% for MRI and 100% for PET-MRI. PET-MRI (median Likert-scale value: 5; range: 4–5) offers a significantly higher reliability of the diagnosis compared to PET (median: 4; range: 2–5; *p* = 0.01) and MRI alone (median: 4; range: 3–5; *p* = 0.03). The number of affected segments in PET-MRI (r_s_ = 0.677; *p* <  0.001) and the MV (r_s_ = 0.703; *p <*  0.001) correlated significantly with the clinical stage. SUVmax (r_s_ = 0.345; *p* = 0.14), SUVpeak (r_s_ = 0.276; *p* = 0.24) and wall thickening (r_s_ = 0.174; *p* = 0.17) did not show a significant correlation to clinical stage.

**Conclusion:**

^18^F-FDG-PET-MRI allows for highly reliable assessment of acute intestinal GvHD and adds information indicating clinical severity.

## Introduction

Allogeneic stem cell transplantation (alloSCT) is increasingly performed in various hematological diseases and remains the most potential curative treatment option in the majority of patients. Graft versus host disease (GvHD) and infections are a major complication of alloSCT, with acute intestinal GvHD contributing to relevant mortality [1]. The three main organs affected by acute GvHD are the skin, liver and gastrointestinal (GI) tract. Their involvement is therefore used in clinical classifications to assess the overall severity of GvHD [2]. Acute intestinal GvHD is typically diagnosed by the assessment of unspecific clinical symptoms (diarrhea, vomiting, abdominal pain, intestinal bleeding), including quantitative stool volume measurements, and exclusion of intestinal infections [2]. Endoscopic evaluation including histopathology has major drawbacks including limited specificity and invasiveness in critically ill alloSCT patients [3], highlighting the need for novel diagnostic strategies [4].

Morphological imaging methods such as contrast-enhanced (CE) computed tomography (CT) and especially magnetic resonance imaging (MRI), as well as metabolic positron emission tomography (PET) imaging are used in the assessment of intestinal inflammation [5–9]. In this context, combined anatomical (MRI) and molecular imaging (^18^F-FDG) proved to be an excellent non-invasive staging method in inflammatory bowel disease, providing different MRI- and PET-derived parameters with high accuracy for the detection of affected segments and correlation to inflammation severity [7]. However, there are only few imaging studies on GvHD, elucidating the role of anatomical CT [10], MRI [11], and PET [8, 12, 13]. First results on the assessment of GvHD by tracing metabolic activity of infiltrating inflammatory cells are promising for recently published preclinical hyperpolarized MRI [14] and for ^18^F-FDG-PET in clinical assessment of patients with intestinal GvHD [8, 12, 13]. CE-MRI also offers a high sensitivity and adds information indicating clinical severity, yet not shown for ^18^F-FDG-PET [11]. Combined PET-MRI may therefore represent a valuable diagnostic tool for the evaluation of intestinal GvHD by combining the advantages of CE MRI and PET, however, there are no reports on the combination of these imaging modalities in intestinal GvHD. The aim of this study was to evaluate the feasibility and the role of ^18^F-FDG-PET-MRI in the diagnosis of acute intestinal GvHD.

## Methods

### Patient population

A cohort of 27 patients with suspected acute intestinal GvHD underwent ^18^F-FDG-PET-MRI between 2/2015 and 8/2019. 6 patients were excluded due to insufficient clinical (*n* = 1) and imaging data (*n* = 3), resection of colon (*n =* 1), and chronic GvHD (*n =* 1). One patient out of this cohort was already published as a case report, focusing on technical aspects of ^18^F FDG-PET-MRI assessment [15]. All patients gave written and informed consent in accordance with the Declaration of Helsinki before PET scan. This study was approved by the local ethics committee (ID: 2020–056-f-S).

### PET-MRI

Examinations were performed on a 3 T Biograph mMR system (Siemens Healthcare). Patients with clinically suspected acute intestinal GvHD were imaged on clinical grounds. All patients were imaged after at least 6 h of fasting and with an adequate blood glucose level (< 150 mg/dL). PET images were acquired 60 min after injection of ^18^F-FDG. Whole-body imaging from the vertex to the mid femur was performed. PET images were acquired for 2 min at each bed position (200 × 200 matrix).

The MRI protocol consisted of a coronal (TE/TR [ms]: 84/1800; field of view (FOV): 380 × 380; matrix: 256 × 320; slice thickness [mm]: 5) and transversal T2 weighted half-Fourier acquisition single-shot turbo spin echo (HASTE) (TE/TR [ms]: 102/1500; FOV: 380 × 420; matrix: 218 × 320; slice thickness [mm]: 6), a coronal (TE/TR [ms]: 1.06/3.16; field of view (FOV): 400 × 450; matrix: 195 × 288; slice thickness [mm]: 3) and transversal 3D T1 volumetric interpolated breath hold examination (VIBE) sequence with fat suppression (TE/TR [ms]: 1.96/4.47; field of view (FOV): 341 × 420; matrix: 182 × 320; slice thickness [mm]: 3) before and after application of contrast agent (Gadovist®; Bayer Healthcare, Leverkusen, Germany).

### Image analysis

The analysis was performed on a per patient basis including the following data sets: PET, MRI and PET-MRI, evaluated by two nuclear medicine readers (W.R., B.N.) in consensus (PET and PET-MRI) and two radiologists (P.S., M.M.) in consensus (MRI and PET-MRI). All readers were aware that patients were referred to ^18^F-FDG-PET-MRI for imaging of suspected intestinal GvHD but were blinded to all other clinical information. The different data sets were graded by the readers according to a five-point Likert scale: 1 = no GvHD, 2 = probably no GvHD, 3 = uncertain, 4 = probably GvHD, 5 = GvHD.

For each data set the number of affected segments was evaluated. For this part of the analysis the intestinal tract was subdivided into 7 segments: duodenum, jejunum, ileum, colon ascendes, colon transversum, colon descendens, sigmoid/rectum.

#### PET analysis

In visual assessment of PET data sets focal or diffusely increased FDG uptake above the background uptake in the intestinal tract and liver uptake was defined as a sign of GvHD [13]. Quantitative PET parameters were assessed using commercially available software (syngo.via, Siemens Healthineers). Standardized Uptake Values (SUV) were measured, reporting the maximal uptake value (SUVmax), and the maximum uptake value in a 1cm^3^ spherical VOI (SUVpeak) in volumes of interest (VOI) encountering parts of the lower gastrointestinal tract with uptake higher than background activity in the gastrointestinal tract and liver uptake. In addition, we defined a threshold of 1.5 x SUVmean liver, inspired by criteria for tumor imaging with ^18^F-FDG-PET, based on reference regions of interest (ROI), placed in the right liver lobe according to established criteria [16]. This threshold was used for defining the metabolic volume (MV) in the VOIs placed in the lower gastrointestinal tract.

Upper parts of the gastrointestinal tract were not evaluated due to high physiological FDG uptake in stomach and pharynx.

#### MRI analysis

MRI parameters assessed to determine the presence of acute intestinal GvHD included mural hyperenhancement, wall thickening, mural stratification and the comb sign [7, 11]. Additional enteric and extra-enteric complications and findings were determined: fistula, abscesses, stenosis and ascites. Stenosis was considered to be present if luminal narrowing was above 50% [11].

### Reference standard

Acute intestinal GvHD was classified following international guidelines [2, 17] estimating the amount of diarrhea per day: stage 0 (< 500 mL/day; < 3 episodes/day), stage I (500–999 mL/day; 3–4 episodes/day): stage II (1000–1500 mL/day; 5–7 episodes/day), stage III (> 1500 mL/day; > 7 episodes/day) and stage IV (severe abdominal pain with or without ileus or grossly bloody stool). Clinical grading was based on clinical stage of skin, liver and intestinal affection of GvHD and was assessed according to current guidelines [2, 17]. A previous study indicated that endoscopy in segmental manifestations of early stage intestinal GvHD is considered to be inferior to PET imaging [12]. Moreover, histopathological findings like cell apoptosis are not specific for intestinal GvHD and a clear diagnosis remains often difficult. Thus, the diagnosis of acute intestinal GvHD was made based on clinical findings including assessment of stool and blood sample screening for viral, bacterial, or fungal gastrointestinal infections to exclude differential diagnosis. In patients where test results and symptoms were not clearly suggestive for intestinal GvHD, further endoscopy examination and evaluation by histopathology were performed. Endoscopic histopathological confirmation of GvHD of the lower gastrointestinal tract by rectosigmoidoscopy or colonoscopy was conducted in 11 patients (52.4%). Confidence levels according to MAGIC consortium were used to assess the reliability of the diagnosis of GvHD in all patients [2]. All available clinical data were reviewed again retrospectively by two experienced hematologists for consistency of the diagnosis of acute intestinal GvHD.

### Statistics

Detection rates for all imaging data sets were calculated assuming that Likert scale values four and five (overall) correspond to a diagnosis of GvHD. A Spearman correlation coefficient was used to measure the strengths of association between PET and MRI and clinical stage and grade. Values of 0.4 ≤ r_s_ <  0.6, 0.6 ≤ r_s_ <  0.8, 0.8 ≤ r_s_ < 1.0 corresponded to moderate, strong and very strong correlation.

For PET, SUVmax, SUVpeak and MV are expressed as mean and 95% confidence intervals. Categorial variables, such as clinical grading of GvHD are presented as absolute and relative frequencies. Between group comparisons of paired variables were tested with Wilcoxon rank sum tests or in case of three groups with a Friedman-test. Multiple comparisons resulted in *p*-value adjustment following Bonferroni’s method. Between group comparisons of unpaired data were tested with Mann-Whitney U.

Results with a *p* <  0.05 were regarded as statistically significant. Analysis was performed using SPSS (version 25.0; IBM SPSS, Somers, NY, USA).

## Results

### Sensitivity and Likert scale

Patients’ characteristics of the 21 patients with suspected acute intestinal GvHD enrolled in this retrospective analysis are shown in Table 1. None of the patients had to be excluded due to laboratory or microbiological testing (e.g. infection with clostridium difficile or CMV).

**Table 1:** Patient characteristics. Abbreviations are listed in the respective section of the manuscript

Characteristic	n	Percentage	Characteristic	n	Percentage
**Number of patients**	21		**ATG**		
**Gender**			no	7	33.3
Female	12	57.1	yes	14	66.7
Male	9	42.9	**conditioning**		
**Age**			reduced dose	9	42.9
	median: 60	range: 33–71	sequential	3	14.3
**Diagnosis**			myeloablative	0	0.0
AML	7	33.3	others	9	42.9
ALL	1	4.8	**immunosuppression**		
CML	1	4.8	CSA/MTX	10	47.6
CLL	0	0.0	CSA/MMF	11	52.4
MDS	5	23.8	other	0	0.0
MPS	1	4.8	**clinical stage**		
MM	1	4.8	0 (< 500)	2	9.5
B-NHL	2	9.5	II(500–1000)	8	38.1
T-NHL	3	14.3	II(1000–1500)	6	28.6
**Match**			III (> 1500)	5	23.8
MRD	8	38.1	IV (blood)	0	0.0
MUD	9	47.6	**Clinical grade**		
MMUD	3	14.3	Grade I	2	9.5
HAPLO	0	0.0	Grade II	8	38.1
**TBI**			Grade III	10	47.6
no	18	85.7	Grade IV	1	4.8
4 Gy	1	4.8	**steroids at date PET**		
8 Gy	2	9.5	yes	14	66.7
12 Gy	0	0.0	no	7	33.3

**Fig. 1:**
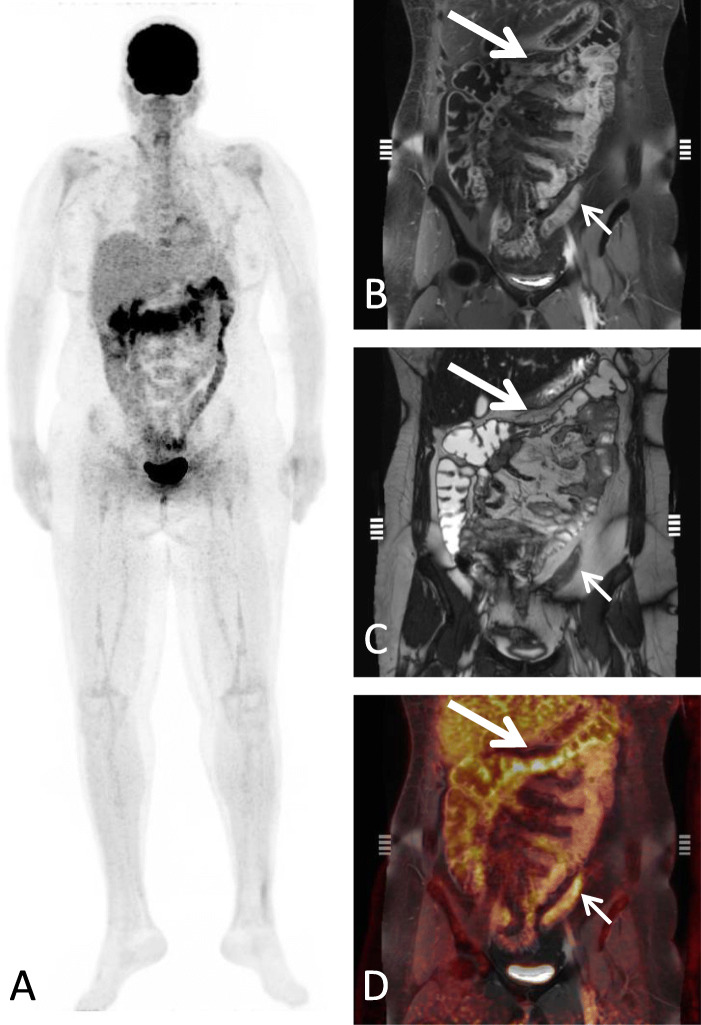
Whole body MIP (A) of an acute intestinal GvHD patient with increased ^18^F-FDG uptake of the colon transversum and descendens. Corresponding coronal MR images reveal mural contrast enhancement of affected large bowel segments (big and small arrow) on T1-weighted contrast enhanced images (B) and stenosis (big arrow) with prestenotic distension as shown on coronal T2-weighted imaging (C) with corresponding increased ^18^F-FDG uptake in fused images (D)

**Fig. 2:**
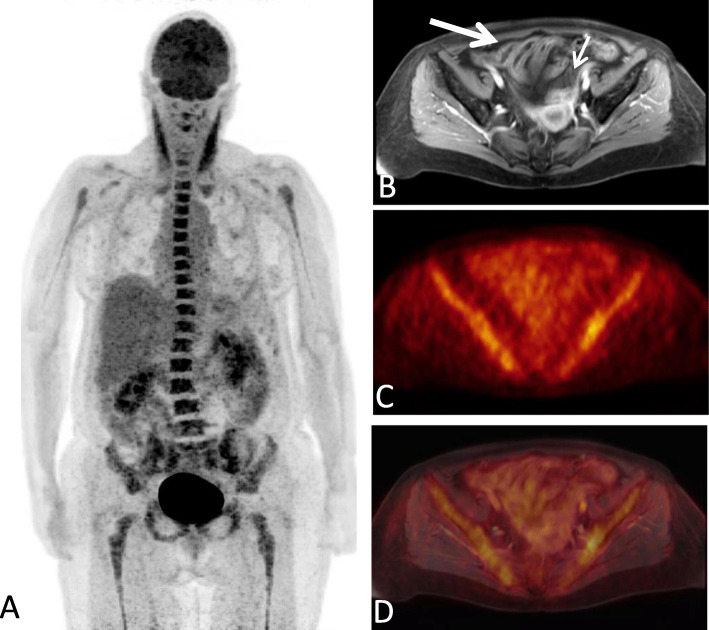
Whole-body MIP (A), contrast enhanced T1 weighted axial MRI sections (B), corresponding PET (C) and fused images (D) of a patient with acute intestinal GvHD (grade II). MR imaging shows contrast enhancement of ileal loops (thick arrow), mural stratification and hypervascular appearance of the mesentery (comb sign) (thin arrow) strongly suggesting presence of acute intestinal GvHD. ^18^F-FDG-PET, however, does not show increased uptake in the affected bowel segments (A,C,D)

Confidence level according to the MAGIC consortium at the date of PET imaging was “confirmed” in eleven patients with biopsy-proven intestinal GvHD and “probable” in the remaining 10 patients, as these were all treated for intestinal GvHD with high-dose corticosteroids as first line treatment. The detection rate for acute intestinal GvHD was 56.8% (95% CI: 32.2–75.6) for PET, 61.4% (95% CI: 36.4–79.3) for MRI and 100% (21/21) for PET-MRI. Significantly different Likert scale values (*p* <  0.001) underlined the higher reliability of imaging-based diagnosis with PET-MR (median: 5; range: 4–5) (Fig. 1) compared to PET (median: 4; range: 2–5; *p* = 0.010) and MRI alone (median: 4; range: 3–5; *p* = 0.03). There was no significant difference between PET and MRI alone regarding the detection rate and Likert scale values. In 4 patients both PET and MRI alone did not detect intestinal GvHD, whereas PET-MRI did. In 9 patients either PET (*n* = 5) or MRI (*n* = 4) were not able to detect intestinal GvHD. Both PET and MRI alone detected intestinal GvHD in 8 patients resulting in a positive detection in PET-MRI as well. One patient with high Likert scale values in MRI compared to PET is depicted in Fig. 2. In the subgroup of patients with initiation of corticosteroid treatment prior to ^18^F-FDG-PET-MRI (*n* = 14), a frequent confounder in^18^F-FDG PET analyses in inflammation, there was a tendency towards a lower detection rate for PET (42.9%) compared to MRI (64.3%) and PET-MRI (100%). In therapy naïve patients (*n* = 7) analysis revealed a detection rate of 85.7% (6/7) for PET and 57.1% (4/7) for MRI.

### PET analysis

**Fig. 3:**
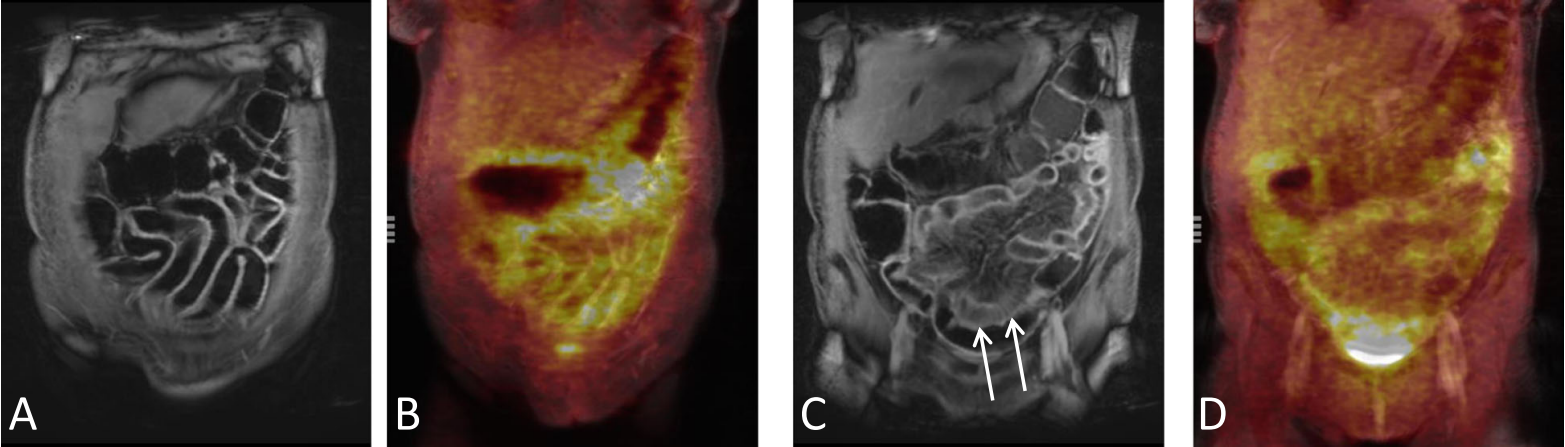
Coronal MR- and fused PET-MRI images of a patient with acute intestinal GvHD. Coronal T1-weighted MRI (A) reveals mural contrast enhancement of the small bowel loops with increased ^18^F-FDG uptake infused images (B). Typical hypervascular appearance of the mesentery (Comb sign) (arrows) adjacent to small bowel loops affected by GvHD (C) with increased ^18^F-FDG uptake in fused images (D)

A single patient showed no elevated tracer accumulation of the intestine and had thus to be excluded from further analysis of quantitative PET parameters (Fig. 2). In another patient, previously published as a case report [15], iron overload of the liver caused an attenuation correction artifact, impeding quantitative uptake measurements for threshold calculation. Thus, this patient was included in measurements of quantitative uptake values of bowel segments but not in MV calculation. PET characteristics of the remaining patients are shown in Table 2. Mean SUVmax and SUVpeak values were above reference liver SUV values (see also Figs. 1 and 3). Mean MV was 274.2 ml (95% CI: 148.8–399.6). The median number of affected segments was four with a range of 1 to 6 segments.

**Table 2:** PET findings of intestinal GvHD patients with positive ^18^F-FDG-PET. One patient was excluded because of negative FDG-PET. An additional patient was excluded from analysis of the metabolic volume due to artefacts impeding reference region measurements

^**18**^F-FDG-PET Parameters	Mean	Range
SUVmax (*n* = 20)	10.5	7.9–13.1
SUVpeak (*n =* 20)	7.6	5.7–9.5
MV [ml] (*n* = 19)	274.2	148.8–399.6

### MRI analysis

Qualitative MRI characteristics are shown in Table 3. All patients presented with wall thickening and mural hyperenhancement (*n* = 21; 100%) with a mean maximal wall thickness of 7.8 ± 1.5 mm (range: 5.5–12.0 mm). Mural stratification (Fig. 2) and comb sign (Fig. 3) could be observed in 57% of patients (*n* = 12). No abscesses or fistulas were reported. Ascites was a common extra-enteric, GvHD-associated finding (*n* = 13; 62%). Only one patient presented with an intestinal stenosis located in the colon transversum (Fig. 1). Median of four segments were affected by intestinal GvHD (range: 1–7 segments).

**Table 3:** MRI findings

MRI Parameters (***n*** = 21 patients)	N	Percentage
Mural hyperenhancement	21/21	100
Mural stratification	12/21	57
Wall thickening	21/21	100
Comb sign	12/21	57
Ascites	13/21	62
Stenosis	1/21	5
Abscess	0/21	0
Fistula	0/21	0

### Correlation with clinical parameters

Correlation between different PET, MRI and PET-MRI parameters and clinical staging are shown in Table 4. The number of affected segments in PET-MRI showed a strong correlation with the clinical stage (r_s_ = 0.677; *p* <  0.001) of acute intestinal GvHD compared to a moderate correlation between the clinical stage and affected segments in PET alone (r_s_ = 0.523; *p* = 0.015), and in MRI alone (r_s_ = 0.540; *p* = 0.011). Interestingly, the metabolic volume MV, assessing the inflamed intestinal tissue volume, showed the highest correlation with the clinical stage (r_s_ = 0.703; *p <*  0.001), while SUVmax (r_s_ = 0.345; *p* = 0.14), SUVpeak (r_s_ = 0.276; *p* = 0.24) and wall thickening (r_s_ = 0.174; *p* = 0.17) did not show any significant correlation to the clinical stage. As patients in these cohort had predominant intestinal affection of GvHD correlation between clinical stage and grade was very strong (r_s_ = 0.944; *p* < 0.001); thus, the correlation of clinical grade and imaging-derived parameters was also highly significant (Table 4). None of the imaging parameters showed significantly different values for patients with “confirmed” intestinal GvHD versus “probable” intestinal GvHD according to the MAGIC consortium criteria (SUV_max_: *p* = 0.710; SUV_peak_: *p* = 0.882; wall thickening: *p* = 0.173; segments PET: *p* = 0.695; segments MRI: *p* = 0.886; segments PET-MRI: *p* = 0.915; MV: *p* = 0.968).

**Table 4:** Correlation of quantitative and semi-quantitative PET/MR parameters and clinical stage of intestinal GvHD

		SUVmax	SUVpeak	wall thickness	segments PET	segments MRI	segments PET-MRI	Metabolic Volume
**Clinical stage**	Spearmen coefficient	0.345	0.276	0.174	0.523	0.540	0.677	0.703
	*p* value	0.136	0.240	0.452	0.015	0.011	< 0.001	< 0.001
**Clinical grade**	Spearmen coefficient	0.372	0.312	0.167	0.474	0.592	0.660	0.692
	p value	0.106	0.181	0.469	0.030	0.014	< 0.001	< 0.001

## Discussion

Intestinal graft-versus-host disease remains a relevant complication after alloSCT, associated with significant morbidity and mortality. To our knowledge, this is the first study to elucidate the feasibility and advantages of a combined ^18^F-FDG-PET-MRI protocol for the diagnosis of intestinal GvHD with special emphasize on quantitative PET and MR parameters and their correlation to clinical stages.

Currently, diagnosis of acute intestinal GvHD includes evaluation of clinical symptoms, stool volume measurements and laboratory testing to exclude differential diagnoses [18]. Endoscopy of the gastrointestinal tract with biopsies has limitations regarding its specificity, revealing apoptotic cells in the mucosa, also observed in other inflammatory conditions [19, 20]. Thus, current guidelines indicate that endoscopy may be helpful to corroborate a clinical impression of possible acute GvHD but is not considered mandatory when alternative diagnoses have been ruled out [21]. Moreover, the procedural risk of endoscopy in these critically ill patients after alloSCT needs to be considered [3]. These limitations highlight the need for novel diagnostic strategies [4]. A recently published study by Assmann et al. makes topical again the potential of visualizing enhanced glycolysis of infiltrating T-cells for the early detection of acute GvHD [14], feasible with metabolic MRI and FDG-PET [22].

In other inflammatory diseases of the lower gastrointestinal tract, such as Crohn’s disease, non-invasive imaging by MRI is already validated as a first line investigation and seen as an alternative to ileocolonoscopy especially in small bowel affection [9]. However, there are only a few studies on the role of MRI in the assessment of intestinal GvHD [11, 23]. In the largest GvHD cohort studied with MRI so far (*n* = 41), Derlin et al. could prove that MR enterography contributes to the detection of intestinal GvHD and adds information to clinical staging [11].

^18^F-FDG-PET has been used to assess intestinal inflammation in chronic inflammatory bowel disease [6], and in a limited number of studies on intestinal GvHD [12, 13]. These studies report a sensitivity for the detection of acute intestinal GvHD of 81–82% [12, 13] for FDG-PET, compared to a sensitivity of 81.5% reported for MR enterography by Derlin et al. [11]. However, individual PET and MRI findings may also be false negative [11, 12]. The lower detection rates for PET and MRI-datasets alone in this study can be explained by the use of a five point Likert scale, allowing for equivocal response, in comparison to dichotomous response assessment in previously published studies, requiring a definite response [11–13]. Interestingly there was a tendency towards higher detection rates of PET in corticosteroid naïve patients in comparison to patients imaged after initiation of corticosteroid treatment, whereas detection rates in MRI were comparable in these two subgroups. This underlines that imaging inflammation on a cellular level with ^18^F-FDG might be more sensitive especially at an early time point of the disease, than looking at changes in morphology and permeability, occurring after immune cell activation. Thus, Bodet-Millin et al. could show that ^18^F-FDG-PET becomes positive even before the development of clinical symptoms [13]. However, this possible advantage in sensitivity is impeded by previous anti-inflammatory treatment regularly observed in patients with intestinal GvHD.

Combining the two imaging modalities FDG-PET and CE-MRI in this study, the detection rate was decisively higher than previously reported for PET [13] and MRI [11] alone. Here, our assumption that combined PET-MR facilitates diagnosis of GvHD compared to PET and MR alone could be quantified by comparison of Likert scale values, being significantly higher for PET-MR compared to PET and MRI alone. In a few patients MRI or PET alone showed no signs of GvHD (Likert scale 1 and 2) or equivocal results (Likert scale 3), while together with the corresponding imaging modality in PET-MRI readers rated the data set as suggestive of GvHD (Likert scale: 4 and 5; see also Fig. 1).

The combination of MRI and ^18^F-FDG-PET in recent studies on PET-MRI in inflammatory bowel disease also proved high accuracy in detecting inflamed bowel segments and correlated well with inflammation severity assessed by endoscopy [7, 24, 25]. Previous PET-MRI studies in inflammatory bowel disease mainly focused on the correlation of quantitative MRI and PET parameters with histological scores on a per segment basis [7, 24, 25]. We have already reported on a correlation between ^18^F-FDG uptake and lymphocyte infiltration in a preclinical model of intestinal GvHD. Translating these results into clinics ^18^F-FDG uptake correlated with GvHD-positive histology in the per segment analysis [12]. New, functional MRI techniques, such as hyperpolarized 13C-pyruvate MRI, allow tracing of glycolysis of infiltrating T-cells, similar to FDG, however only in a distinct target organ, not allowing for whole body imaging [14].

To our knowledge we are the first to report on the assessment of acute intestinal GvHD with a PET-MRI protocol, focusing on the correlation of different PET and MRI-parameters, comparable to previous PET-MRI studies on inflammatory bowel disease [7, 24], to clinical stage of GvHD. Similarly to Derlin et al. [11] and Budjan et al. [23], mural hyperenhancement and wall thickening were common findings in our patient cohort. However, in accordance with prior results [11] MRI wall thickening is not able to predict the clinical stage and grade. The most common extra-enteric criteria was ascites. Abscesses and fistulas, more common in chronic inflammatory conditions of the bowel, were not reported in previous studies just as in ours [11].

On a per patient basis frequently used SUVmax does not correlate significantly to clinical stage and grade in this study. These results are in line with data presented by Bodet-Milin et al. [13]. Assessing the number of involved bowel segments allows for a significant correlation to clinical stage and grade, as previously shown for the number of affected segments in MRI [11] and CE-CT [26]. Interestingly and new for assessing the volume of intestinal inflammation by PET, the quantitative PET parameter metabolic volume revealed the highest correlation with clinical stage and grade. As patients included in this analysis presented with predominant intestinal affection of GvHD, clinical stage and grade showed a very strong correlation. One of the limitations of this study is that neither spasmolytics nor oral contrast agents or bowel purgation were regularly used being in line with previously published results on MR enterography in intestinal GvHD [11]. Previous bowel purgation might impede correlation between ^18^F-FDG-PET and histological inflammatory grading and is considered as burdensome [24]. Oral intake of fluid prior to ^18^F-FDG-PET included in many study protocols [7, 24, 25] might additionally alter the motility of the bowel, influencing ^18^F-FDG uptake and quantitative MRI parameter [24].

Diffusion-weighted imaging (DWI) of the abdomen, often included in prospective studies on inflammatory bowel disease, was not included in this retrospective analysis, as it was only part of the clinical protocol in 4 patients. Results on the role of ADC values in comparison to quantitative PET parameters are heterogeneous with higher [7, 25], or similar [24] accuracy of SUVmax for the detection of inflammatory bowel segments. The role of ADC values for the correlation to clinical parameters on a per patient basis was not evaluated yet, however, might be comparable to quantitative PET parameter SUVmax and SUVpeak.

The main limitations of this study are its low patient number, its retrospective design and the inhomogeneity of imaging time points in relation to the initiation of immunosuppressive therapy. Due to the retrospective study design and the dedicated abdominal MR-imaging protocol in this study no ^18^F-FDG-PET-MRI control group could be included. Accuracy could not be calculated on a per patient basis in the present study, as there were no patients without intestinal GvHD in this PET-MRI cohort. Another aforementioned limitation is the lack of serial or targeted biopsies throughout the intestinal tract, impeding a per segment analysis in these severely ill patients after alloSCT. However, the value of histopathological confirmation in the diagnosis of GvHD remains a point of discussion, even in the era of advanced imaging and other biomarkers [1, 14].

## Conclusion

In summary, combined ^18^F-FDG-PET-MRI is an accurate non-invasive tool for the assessment of intestinal GvHD, offering a higher sensitivity compared to morphological and metabolic imaging alone. The number of affected segments as assessed by ^18^F-FDG-PET-MRI and especially the ^18^F-FDG-derived metabolic/inflammatory volume add diagnostic information, since both parameters correlated with the clinical severity of intestinal GvHD.

## Data Availability

Analyzed data are stored at the Department of Nuclear medicine, University hospital Münster, Germany and are available from the corresponding author on reasonable request if not already included in this article.

## Abbreviations

GvHD: Graft versus host disease
alloSCT: Allogeneic stem cell transplantation
MV: Metabolic volume
CE: Contrast-enhanced
SUV: Standardized Uptake Values
ROI: Regions of interest
CT: Computed tomography
PET: Positron emission tomography
MRI: Magnetic resonance imaging
AML: Acute myeloid leukemia
ALL: Acute lymphocytic leukemia
CML: Chronic myeloid leukemia
CLL: Chronic lymphocytic leukemia
MDS: Myelodysplastic syndrome
MPS: Myeloproliferative syndrome
MM: Multiple myeloma
NHL: Non Hodgkin Lymphoma
MRD: Matched-related donor
MUD: Matched-unrelated donor
MMUD: Mismatched-unrelated donor
HAPLO: Haploidentical
ATG: Anti-thymocyte-globulin
TBI: Total body irradiation
CSA: Cyclosporine A
MTX: Metotrexate
MMF: Mycofenolate mofetil
